# Sequencing of bulks of segregants allows dissection of genetic control of amylose content in rice

**DOI:** 10.1111/pbi.12752

**Published:** 2017-07-11

**Authors:** Peterson Wambugu, Marie‐Noelle Ndjiondjop, Agnelo Furtado, Robert Henry

**Affiliations:** ^1^ Queensland Alliance for Agriculture and Food Innovation University of Queensland Brisbane Qld Australia; ^2^ Africa Rice Center (AfricaRice) Cotonou Benin; ^3^ Present address: Kenya Agricultural and Livestock Research Organization (KALRO) Genetic Resources Research Institute Nairobi Kenya

**Keywords:** amylose content, bulk segregant analysis, genetic markers, genetic linkage, allele frequency, whole genome sequencing

## Abstract

Amylose content (AC) is a key quality trait in rice. A cross between *Oryza glaberrima* (African rice) and *Oryza sativa* (Asian rice) segregating for AC was analysed by sequencing bulks of individuals with high and low AC. SNP associated with the *granule bound starch synthase (GBSS1)* locus on chromosome 6 were polymorphic between the bulks. In particular, a G/A SNP that would result in an Asp to Asn mutation was identified. This amino acid substitution may be responsible for differences in *GBSS* activity as it is adjacent to a disulphide linkage conserved in all grass *GBSS* proteins. Other polymorphisms in genomic regions closely surrounding this variation may be the result of linkage drag. In addition to the variant in the starch biosynthesis gene, SNP on chromosomes 1 and 11 linked to AC was also identified. SNP was found in the genes encoding the *NAC* and *CCAAT‐HAP5* transcription factors that have previously been linked to starch biosynthesis. This study has demonstrated that the approach of sequencing bulks was able to identify genes on different chromosomes associated with this complex trait.

## Introduction

*Oryza glaberrima*, commonly referred to as African rice, is one of the two independently domesticated rice species, the other one being *Oryza sativa,* which is also commonly referred to as Asian rice. African rice is a valuable source of genetic diversity for rice improvement. Its genetic potential in terms of resistance/tolerance to biotic and abiotic stresses has been well documented and deployed in rice improvement (Wambugu *et al*., 2013). Recently, it has been reported to have some unique starch traits which could confer potential health benefits among them being higher amylose content (AC) and gelatinization temperature than Asian rice (Wang *et al*., 2015). AC is a major determinant of the cooking, eating and milling properties of rice. High AC has been associated with nutritional and health benefits by having a positive effect on various indices of gastrointestinal health. The high AC and gelatinization temperature are likely to result in decreased digestion rate thereby making African rice a potential natural source of slowly digestible starch (Wang *et al*., 2015). With the health benefits of high amylose foods increasingly getting recognized, African rice seems to be a valuable genetic resource for breeding premium‐value genotypes with high AC, preferably in high yielding and elite genetic backgrounds. Though high amylose foods generally have increased resistant starch, the AC in African rice may not be high enough to influence the resistant starch component.

Elevated levels of AC can be achieved by altering the activity of various enzymes especially those involved in starch elongation and branching (Regina *et al*., 2014). The various mechanisms through which this can be achieved include increasing the activity of GBSS1 and reducing amylopectin synthesis by suppressing the activity of *starch synthases* and *starch branching enzymes (SBE)* (Butardo *et al*., 2011; Regina *et al*., 2006; Zhang *et al*., 2011). However, the use of mutagenesis and genetic engineering in producing starch with higher AC than is naturally found in rice, is associated with undesirable traits (Butardo *et al*., 2016; Liu *et al*., 2014; Nandkishor, 2015). The use of naturally occurring variation in breeding for amylose therefore remains the most preferred option. Though many methods used in determination of AC are accurate and relatively easy to use, majority of them are costly in terms of consumables and equipment (Delwiche *et al*., 1995; Hu *et al*., 2015). The use of marker‐assisted selection (MAS) would be important in breeding for amylose as it can help in speedy and cost‐effective delivery of improved varieties possessing superior grain quality traits.

However, the lack of adequate information on the genetic basis of some starch properties has been a hindrance in selecting for them (Bao *et al*., 2006). AC is known to be primarily regulated by *granule bound starch synthase (GBSS1),* with several transcription factors (TFs) being reported to be involved in the transcriptional and post‐transcriptional regulation of this starch gene. Among these include *OsBP‐5*, a MYC TF (Zhu *et al.,* 2003) and *OsbZIP58*, a basic leucine zipper (bZIP) TF (Wang *et al*., 2013) both of which are key regulators of amylose synthesis. Recently, a genomewide systems genetics study identified a bHLH TF as being involved in determining the proportion of AC perhaps by its role in the transcriptional regulation of GBSS1 (Butardo *et al*., 2016). Despite this increased understanding of the genetic mechanisms underlying amylose synthesis particularly at the transcriptional level, this pathway to a great extent remains poorly understood (Wang *et al*., 2013).While several functional nucleotide polymorphisms have been found to associate with AC in Asian rice, (Ayres *et al*., 1997; Bligh *et al*., 1995; Dobo *et al*., 2010; Larkin and Park, 2003), no marker‐trait associations have been identified in African rice. This lack of this information acts as a major hindrance to the application of MAS.

Previous studies on the genetic architecture of AC in African rice have analysed the various individual loci in isolation rather than conducting global genome analyses. This locus‐specific approach may have led to the poor understanding of the genetic mechanism underlying this trait. This study therefore set out to dissect the genetic architecture of AC in African rice by conducting a genomewide analysis of any underlying candidate genes and genetic variants. Leveraging the current advances in genomics, this study used an integrative genomics approach to conduct genetic mapping of the backcross progenies of an interspecific cross between *O. sativa* and *O. glaberrima*. This integrative approach involved whole genome‐based bulk segregant analysis (BSA) (Michelmore *et al*., 1991) as well as gene co‐expression and gene regulatory analysis. Next generation sequencing (NGS) aided BSA, commonly referred as mapping by sequencing (Hartwig *et al*., 2012), provides a robust genetic mapping approach that has successfully been used in identifying causal mutations and candidate genes in various crops among them tomato (Illa‐Berenguer *et al*., 2015), sorghum (Han *et al*., 2015), chickpea (Das *et al*., 2015), cucumber (Lu *et al*., 2014), rice (Takagi *et al*., 2013; Yang *et al*., 2013), sugar beet (Ries *et al*., 2016) and pigeon pea (Singh *et al*., 2015). Analysis of polymorphisms between the bulks identified SNPs associated with *GBSS1*, with a G/A SNP located in exon 12 potentially being linked with AC in African rice. Two candidate TFs that may be involved in transcriptional regulation of *GBSS1* structural gene were identified. Our study provides useful insights on genetic control of AC and forms a useful follow‐up to the study that was recently published by Butardo *et al*. (2016).

## Results

### Analysis of amylose content and identification of pool segregants

**Figure 1:**
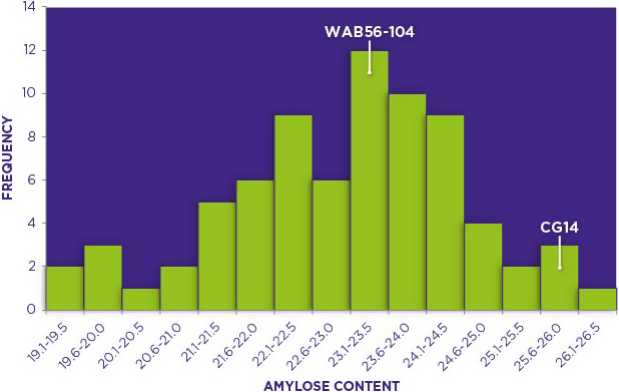
Frequency distribution of amylose content (AC) in 100 individuals and parental genotypes of a BC_2_F_8_ population of an interspecific cross between *Oryza glaberrima* and *Oryza sativa* (CG14 X WAB 56–104). AC was determined using the Megazyme kit as a % of total starch by weight.

Phenotyping for AC was carried out on a total of 100 progenies of an interspecific cross between African rice and *O. sativa*. This phenotypic analysis resulted in a normal distribution with the AC ranging from 19.2% to 26.2% in the interspecific progenies. CG14, the African rice parent had AC of 25.7% while that of WAB 56–104, the *O. sativa* parent, was 23.2% thereby showing that there was transgressive segregation on both sides of the phenotypic distribution. This transgressive segregation was largely skewed towards the direction of the low amylose parent (Figure 1), perhaps due to the numerous backcrosses conducted towards this parent. Based on the phenotypic analysis data, individual progenies to be used in constituting the two phenotypically distinct amylose bulks to be in BSA were selected. The two bulks were selected by pooling individuals on the extreme ends of the phenotypic distribution, with each of the two bulks having ten individuals. The low amylose bulk (LAB) had segregants with amylose ranging from 19.2% to 21.6% while those in the high amylose bulk (HAB) ranged from 24.1% to 26.2%.

### DNA sequencing and mapping

The pooled DNA together with that of the parents was subjected to whole genome sequencing. Sequencing the two phenotype‐based DNA pools generated a total of 131 008 138 and 145 429 179 paired end reads for the high and LAB s, respectively (Table S1). The mean sequencing depths were 53X and 59X for the HAB and LAB, respectively. The trimmed reads were separately mapped to reference genome sequences of both African and Asian rice. A total of 112 021 886 and 119 224 875 reads were uniquely mapped to the *O. sativa* reference, yielding average nuclear genome coverages of about 85% and 81% for HAB and LAB, respectively (Table S2).

Variant calling resulted in a total of 280 875 and 333 299 SNPs for the high and low bulks, respectively, when mapped to the *O. sativa* reference. As expected, mapping to the *O. glaberrima* reference resulted in a significantly lower number of SNPs for the HAB, totalling 88 748 while the low bulk had a significantly higher number of SNPs, totalling 391 032 (Table 1). This significant difference in the number of variants between the two bulks when mapped to the two references is due to the fact that the high bulk included many individuals with a higher proportion of the *O. glaberrima* genome than the low bulk. On the other hand, the low bulk consisted of many individuals with a higher proportion of the *O. sativa* genome than the high bulk. As the *O. sativa* reference sequence (IRGSP, 2005) has higher quality annotations than African rice reference (Wang *et al*., 2014), further analysis was conducted on the variants called using the *O. sativa* nipponbare reference.

**Table 1:** Number of SNPs called for the high and LAB and two parental genomes when mapped to *Oryza sativa* and *Oryza glaberrima* references

Sample	*O. sativa* reference	*O. glaberrima* reference
HAB	280 875	88 748
LAB	333 299	391 032
WAB 56–104	594 358	1 802 859
CG14	2 329 714	4145

HAB, high amylose bulk; LAB, low amylose bulk.

### Association analysis identifies putative amylose‐linked genetic markers

**Figure 2:**
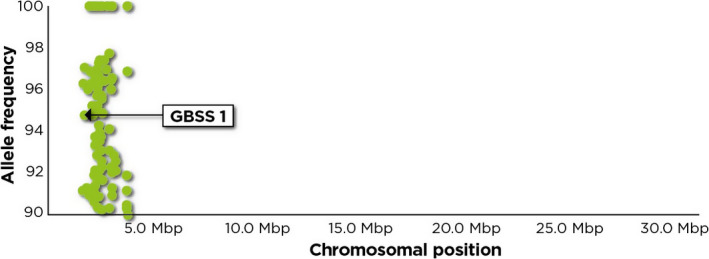
Plot of chromosomal positions and allele frequency of putative candidate nonsynonymous SNPs located on chromosome 6. The indicated allele frequencies are from the high amylose bulk. Almost all the putative candidate genes where these SNPs are located, cluster with *GBSS1* suggesting an apparent close linkage with this locus. The putative candidate genes cluster within a region of about 2.1 Mbp which is equivalent to about 10 cM. The SNPs were plotted using CandiSNP (Etherington *et al*., 2014).

Genomewide analysis of polymorphisms between the two phenotypically distinct bulks led to the identification of several amylose‐linked SNPs that are associated with granule bound starch synthase (*GBSS1*). These include two SNPs that are located in the 5′ UTR and 2 nonsynonymous SNPs located in the exonic regions (Table 2). A nonsynonymous G/A SNP associated with *GBSS1* that would result in an Asp to Asn mutation was identified. This conservative amino acid change is located on chromosome 12 of African rice. The other nonsynonymous SNP is an A/C SNP located on exon 6 which is a well‐known amylose‐linked SNP. This SNP has only been reported in *O. sativa* and appears to be fixed in *O. glaberrima*. Other amylose‐linked polymorphisms were found in genomic regions surrounding *GBSS1*. These polymorphisms which lie near the terminal region of the short arm of chromosome 6, cluster in a genomic region of approximately 2.1 Mbp, covering chromosomal position 1 653 659 bp to 3 794 599 bp (Figure 2 and Table S3).

**Table 2:** Upstream and nonsynonymous SNPs putatively linked to amylose content identified in candidate genes

Gene name	Loci	SNP	Region	Location	Amino acid change
*GBSS1*	LOC_Os06g04200	G/A	Exon	1 769 686	Asp to Asn
*GBSS1*	LOC_Os06g04200	A/C	Exon	1 768 006	Tyr to Ser
*GBSS1*	LOC_Os06g04200	G/A	5′UTR	1 765 976	N/A
*GBSS1*	LOC_Os06g04200	C/T	5′UTR	1 766 005	N/A
*NAC*	LOC_Os11g31330	G/A	Exon	18 288 616	Val to Ile
*Glutelin subunit mRNA*	LOC_Os01g55690	C/A	Exon	32 077 903	Leu to Met
*Glutelin subunit mRNA*	LOC_Os01g55690	G/A	Exon	32 078 006	Ser to Asn
*Glutelin*	LOC_Os01g55630	C/T	Exon	32 053 231	Ile to Thr
*Basic helix‐loop‐helix (OsbHLH071)*	LOC_Os01g01600	T/C	Exon	304 355	Leu to Pro
*Starch synthase IVa*	LOC_Os01g52250	T/A	Exon	30 038 502	Ser to Thr
Histone‐fold domain‐containing protein	LOC_Os01g01290		Exon	141 117	

In addition to the variants located on chromosome 6, other amylose‐linked SNPs were found on chromosome 1 and 11. Some of the amylose‐linked SNPs located outside chromosome 6 were found in genes that have previously been associated with starch biosynthesis. These include *starch synthase IVa* (LOC_Os01g52250), Histone‐fold domain‐containing protein (LOC_Os01g01290) both of which are located on chromosome 1 and a *NAC* (for NAM, ATAF, and CUC) TF located on chromosome 11 (Table 2). Other candidate genes with no known role in starch biosynthesis include *glutelin subunit mRNA* (LOC_Os01g55690) and *basic helix‐loop‐helix* (OsbHLH071) located on chromosome 1.

The identified marker sites had the alleles from the high amylose parent significantly over‐represented in the HAB. Under the BSA approach, it would be expected that approximately 50% of the reads in loci that have no genetic effect on the trait of interest would be derived from each of the two parental genomes. On the other hand, allele frequency would differ noticeably in genomic regions controlling the trait, with such regions showing significant over‐representation of reads from one of the parental genomes.

Studies have indicated that very low coverage in genetic marker sites may lead to sampling error which may cause spurious deviations in allele frequency from the expected 50% segregation pattern in regions not linked to the phenotype (Duitama *et al*., 2014). To weed out false‐positive associations, read coverage was therefore assessed across the genome and also specifically in the identified candidate genetic marker sites. Analysis of coverage in individual SNP positions indicated that it ranged from 21 to 41.

### Gene expression profiles and gene co‐expression analysis

Gene co‐expression analysis is based on the assumption that genes with similar expression profiles are likely to have some functional linkage. It has proven to be a powerful approach in the identification of candidate genes and TFs regulating various biological processes, among them starch biosynthesis in rice (Aoki *et al.,* 2007; Fu and Xue, 2010). Gene co‐expression analysis of the identified amylose candidate genes was conducted using known starch biosynthesis genes as guide genes. Analysing the identified candidate genes using this approach showed that genes encoding both *NAC* (LOC_Os11g31330) and *CCAAT_HAP5* (LOC_Os01g01290) TFs were co‐expressed with various starch genes. *NAC* TF was co‐expressed with six starch biosynthesis genes among them being *GBSS1* and *MADS29*, both of which are involved in regulating amylose biosynthesis. Similarly, *CCAAT*_HAP5 TF was also co‐expressed with *GBSS1* among other starch genes (Table 3). Most of the candidate genes had high endospermic gene expression thus exhibiting patterns similar to that of *GBSS1* (Figures S1–S3).

**Table 3:** Amylose candidate genes/transcription factors (TF) showing co‐expression with known starch biosynthesis genes

Gene/TF		Co‐expressed starch genes	Weighed Pearson correlation coefficient for gene co‐expression
Locus ID	Name		
LOC_Os01g01290	Histone‐fold domain‐containing protein	Granule‐bound starch synthase 1	0.709491
		Starch branching enzyme 1	0.690723
		ADP‐glucose pyrophosphorylase subunit SH2	0.725921
LOC_Os11g31330	*NAC*	ADP‐glucose pyrophosphorylase large subunit 2	0.748331
		Starch branching enzyme 3	0.732204
		Pullulanase	0.636763
		Granule‐bound starch synthase 1	0.785246
		MADS29	0.83
		Starch branching enzyme 1	0.744933

### Analysis of conserved regulatory motifs

Conserved *cis*‐regulatory motifs in the candidate genes were analysed using the RiceFREND platform (Sato *et al.,* 2011). Some of regulatory motifs that were present in the candidate genes are shown in Table S5. Notably, conserved motifs that confer seed and endosperm‐specific gene expression were among them. Previous studies have shown that the ACGT elements are important in the transcriptional regulation of starch genes (Wang *et al*., 2013). Analysis of the candidate genes shows that both *NAC* and *CCAAT‐HAP*5 shared these ACGT core elements with *GBSS1*. Key starch biosynthesis genes among them *GBSS1*,* starch branching enzyme* and *pullulanase*, all of which showed similar co‐expression profiles with the candidate genes, have the ACGT core motif. Both *GBSS1* and *CCAAT‐HAP* TF possess the CCAAT‐box. Also present were motifs related to regulation of seed protein storage genes particularly those involved in glutelin synthesis.

### Allele mining in natural variation of African rice *GBSS1* reveals novel alleles

Biparental populations segregate for only a fraction of the total variation available in a species. As functional variation in *GBSS1* appeared to be limited in the interspecific cross, we analysed the diversity available in *GBSS1* in a diverse set of unrelated *O. glaberrima* accessions. Whole genome short reads of ten African rice accessions were mapped to the *GBSS1* gene from *O. sativa* with variant calling resulting in five novel putative SNPs spread across several exons (Table 4). The majority of the identified SNPs seem to be of low frequency, with three of them appearing in only one of the ten accessions studied.

**Table 4:** Putative SNPs found in *GBSS1* from African rice

Position^a^	Exon	SNP	Amino acid change
139	2	T/G	Ser to Ala
250	2	A/C	Asn to His
1247	10	A/C	Asp to Ala
1272	10	G/C	Glu to Asp
1582^b^	12	G/A	Asp to Asn

a Positions are numbered from the translation start codon.

b This G/A SNP has previously been identified but its functional role has not been elucidated.

## Discussion

### Genetics of amylose content

Despite African rice having potential for contributing genes to improve rice quality (Wambugu *et al*., 2013), research on starch structure and properties as well as the genetic mechanisms controlling them have for a long time been neglected in this cultivated species. Interest in studying starch‐related traits in African rice is however growing (Gayin *et al*., 2015, 2016a,b; Wang *et al*., 2015). This study investigated the genetic basis of AC in African rice by genetic mapping of the progenies of an interspecific cross between *O. glaberrima* and *O. sativa*. The continuous variation and normal distribution of AC observed in this study has been reported in previous studies (Aluko *et al*., 2004) and indicates that AC in African rice is quantitatively inherited. The normal distribution suggests that amylose is under polygenic control. The AC is determined not only by the activity of *GBSS1* but also by the relative activity of the amylopectin synthesizing enzymes namely *starch synthases* and *starch branching enzymes*. *ADP‐glucose pyrophosphorylase (AGPase) and pullulanase* also play minor roles in amylose synthesis (Tian *et al*., 2009; Sun *et al*., 2011). Other studies have reported amylose to be under the control of other loci in addition to the waxy locus though majority of these loci have largely remained unidentified (Aluko *et al*., 2004; He *et al*., 1999). Identification of these additional candidate genes remains an important area of research focus as these genes are likely to provide new targets for AC modification. The transgressive segregants observed in this study indicate that there exists potential of genetic improvement of AC in these two cultivated species.

### Bulk segregant analysis identifies major and minor loci associated with amylose content

SNPs were found in genes known to influence amylose biosynthesis as well as some which have previously been linked with starch biosynthesis but whose role in amylose synthesis had not been established. *GBSS1* is a major effect loci explaining as high as 90% of the AC variation in some genetic backgrounds (He *et al*., 1999). Among the markers identified in this study were four SNPs found in the exonic and promoter regions of *GBSS1* structural gene. These include a nonsynonymous G/A SNP located in exon 12 and which remains the only SNP that has been reported by previous studies in African rice to date. This SNP leads to a change from Asparagine to Aspartic acid which is predicted to be a conservative amino acid change (Dobo, 2006; Umeda *et al*., 1991). Though this SNP leads to a conservative amino acid change, it can be functionally important if located near an enzyme active site (Henry, 2008).

This SNP affects the *GBSS1* protein at position 528. Analysis of the three‐dimensional structure of *GBSS1* protein (Momma and Fujimoto, 2012) shows that residue 528 is in the glycosyltransferase region of the protein and is adjacent to a disulphide link which is conserved in the Poaceae but not in other species. A change from aspartic acid in *O. sativa* to asparagine in *O. glaberrima* at position 528 could influence the stability of the S‐S link and thereby affect *GBSS1* activity. A similar argument has been advanced for the A/C SNP located on chromosome 6 which causes a serine/tyrosine amino acid substitution in *O. sativa*. This SNP influences AC by destabilizing *GBSS1* through a hydrogen bonding, hence affecting its activity (Dobo *et al*., 2010; Pace *et al*., 2001). Functional characterization of the G/A SNP through targeted mutagenesis is recommended, as this would help clearly elucidate on functional importance of this SNP. Relating to the two SNPs found in the promoter of *GBSS1*, it is possible that they may have some regulatory role in amylose synthesis.

In addition to *GBSS1*, we identified other amylose candidate genes and markers, among which include TFs in the *NAC* (LOC_Os11g31330) and *CCAAT‐HAP5* (LOC_Os01g01290) families. Though both of these TFs have previously been reported to be co‐expressed with starch synthesis genes showing preferential expression in both the endosperm and vegetative tissues (Fu and Xue, 2010), their specific role in starch biosynthesis is however unknown. *NAC* TFs form one of the largest group of plant‐specific TFs, with over 150 of these having been identified in rice (Nuruzzaman *et al.,* 2010). They have been widely reported to be involved in the control of leaf senescence and are involved in the remobilization of nutrients into the developing seed (Sperotto *et al.,* 2009). In wheat, a *NAC* homologue (*NAM‐B1*) was found to cause increased protein content, zinc and iron by accelerating senescence which subsequently increased nutrient remobilization (Uauy *et al.,* 2006). Though there appears to be no documented role of *NAC* in starch synthesis, nutrient remobilization may potentially lead to an increase in starch content.

Three SNPs found in two endosperm‐specific genes which are involved in glutelin synthesis were identified. Identification of SNPs located in genes involved in glutelin synthesis suggests a close interaction between the starch biosynthesis and glutelin synthesis pathways. This is consistent with results of a recent study which reported that there exists a link between glutelin and starch physicochemical properties in rice (Baxter *et al*., 2014). These authors reported a positive correlation between glutelin content and hardness as well as adhesive properties of starch. It is important to note that the relationship between AC and hardness is well known, with high amylose being associated with hard texture. It is therefore possible that glutelin has a direct correlation with AC and in turn affecting other physicochemical properties such as hardness.

### Gene co‐expression analysis helps identify functionally related genes

This study used an integrative genetics approach that employed a combination of genetic mapping strategies to enhance the discovery and use of useful genetic variation. Among the approaches used is gene co‐expression analysis which has successfully been used in identifying regulatory factors involved in various metabolic pathways. The two candidate TFs that were found to be putatively associated with amylose were co‐expressed with various starch genes (Table 3). The major starch genes that were co‐expressed with these candidates TFs include *GBSS1, ADP‐glucose pyrophosphorylase, starch branching enzymes* and *pullulanase*. This perhaps suggests that these starch genes might have some role in regulating amylose synthesis. This is consistent with the findings of Tian *et al*. (2009) and Sun *et al*. (2011) who reported that *ADP‐glucose pyrophosphorylase (AGPase), pullulanase, SSIIIa, starch branching enzyme 1 (SBE1)* and *SSI* play minor roles in amylose synthesis by interacting with *GBSS1*. These starch genes were however not functionally associated with amylose in the mapping population studied here.

The *NAC* TF is co‐expressed with six starch biosynthesis genes (Table 3). Co‐expression with such a high number of starch genes might be an indicator of the functional importance of this TF in the starch biosynthesis pathway. Among the genes with which it shows co‐expression patterns include *GBSS1* and *MADS29*, both of which have roles in amylose biosynthesis. The *CCAAT_HAP5* TF shares the CCAAT‐box with *GBSS1* and is co‐expressed with *GBSS1*,* SBEI* and *ADP‐glucose pyrophosphorylase subunit SH2*. These gene co‐expression profiles act as a further pointer of the potential functional involvement of these TFs in amylose biosynthesis. Co‐expression of *NAC* TF and *MADS29* suggests that they may have the same transcription regulatory mechanisms in starch synthesis. Though gene co‐expression analysis is a powerful approach in predicting gene function gene, it can be noisy and may not necessarily indicate functionally relevant relationships (Hansen *et al.,* 2014; Yeung *et al.,* 2004). Results of gene co‐expression analysis may need to be interpreted with caution. However, combining gene co‐expression with BSA helped improve the reliability of our results.

### Analysis of allele frequency detects linkage drag around waxy locus

One of the challenges that faces genetic mapping efforts and specifically those aimed at identifying trait causative mutations is distinguishing truly linked and spuriously linked genomic regions. The clustering of majority of the candidate markers in a genomic region of about 2.1 Mbp could be as a result of linkage drag. A recent study by Butardo *et al*. (2016) however shows that some of the genes identified in this genomic region may be functionally associated with AC. These authors found *WASH complex subunit 7‐like isoform X1* (LOC_Os06g04520) which is among the genes located in this genomic region to be differentially expressed and to be associated with AC. However, although this study found this gene to have some association with AC, we identified a different SNP from the one reported by Butardo *et al*. (2016) as being linked with AC.

Linkage causes markers surrounding the causal loci to show deviations from the 50% inheritance pattern expected of parental alleles, though they may not be linked to the trait (Duitama *et al*., 2014). Genetic linkage around the waxy locus has previously been reported (Heuer and Miézan, 2003; Sano, 1990). A cluster of more than 15 QTLs associated with different traits has been found in the region encompassing the 2.1 Mbp genomic interval identified in the present study (Yonemaru *et al*., 2010). The presence of such a large number of QTLs is most likely due to linkage of different genes controlling different traits. With the causal mutation, possibly embedded in a large genomic region, the challenge in the hands of molecular geneticists is how to narrow down this region so as to identify the causal mutation. Markers showing linkage with the causal loci have potential for universal application in marker‐assisted breeding and are especially useful in cases where there is no reliable marker for genotyping the causal loci (Kim *et al*., 2015). This might be useful in the case of African rice where polymorphisms in *GBSS1* are limited as observed in the interspecific cross. The linked markers located in the 2.1 Mbp region might be vital resources that can potentially be used to indirectly select for amylose in a practical breeding programme. The linkage drag observed in this study on the other hand has potential for presenting challenges to breeders due to the possible co‐introduction of desirable alleles together with those associated with poor traits.

### Potential of bulk segregant analysis in genetic mapping

With only a few studies having employed whole genome‐based BSA, the potential for this state of the art genomic approach is only starting to be revealed. BSA was initially designed to target major effect genes however its continued advances has increased its resolution to detect many underlying genetic factors including minor causal alleles (Ian *et al*., 2010; Venuprasad *et al*., 2009; Vikram *et al*., 2012; Zou *et al*., 2016). While it has been reported that using initial populations of 100–300 individuals only allows the identification of major loci (Sun *et al*., 2010), results of this study seem to contradict this. With an initial population of 100 progenies, this study identified *GBSS1* and several others as being linked to amylose, thus demonstrating the potential of this approach in identifying major and minor loci associated with complex traits. The high resolution observed in this study even with seemingly low initial population size might be explained by marker density as sequencing the whole genome provided the highest density of markers possible. Moreover, the use of recombinant inbred lines (RILs) (BC_2_F_8_) may also have contributed to the increased resolution. In a simulation study, Takagi *et al*. (2013) found that using RIL gives more power in detecting QTLs than F2 population.

We conducted deep sequencing, achieving average coverages of 35X and 38X of reads uniquely mapped against the Nipponbare reference for both high amylose and LABs, respectively (Table S2). This deep coverage enabled sequencing of all the chromosomes in the pooled segregants in addition to making it possible to distinguish rare variants from sequencing errors. Analysis of coverage in individual candidate marker positions shows that all of them had high coverage which ranged from 21 to 41. This coverage ruled out the possibility of spurious deviations in allele frequency from the expected 50% segregation pattern due to low coverage, which could have led these markers/region being falsely linked to amylose (Duitama *et al*., 2014).

### Desirable attributes of African rice

The eating and cooking properties of African rice are poorly studied. Until clinical data are available, the real benefits of the higher AC will remain unknown. However, anecdotal reports from consumers of African rice suggest that it is less digestible and provides more postmeal satiety than Asian rice, an attribute that makes it highly preferred in West Africa (Linares, 2002; Nuijten *et al*., 2013; Teeken *et al*., 2012). Though these consumers contend than Asian rice is sweeter than African rice, they report that the former is ‘lighter’ and provides less satiety than African rice. Due to these reasons, Asian rice tends to be consumed at higher rates than African rice. To reduce the consumption rates of Asian rice, consumers mix the two types of rice and then cook them together (Teeken *et al.,* 2011). This practice presumably helps to slow the digestion rate of Asian rice and has the overall effect of reducing the amount of carbohydrates consumed per serving. This in turn has a twofold effect; on one hand, reducing carbohydrate intake leads to reduced risks of type 2 diabetes (Sheard *et al*., 2004) and on the other hand it has positive food security implications as households are able to save some food for the future. This provides an interesting perspective to food security as improving the intrinsic properties and value of food especially among the poor communities as a way of addressing food insecurity is rarely considered. High AC has been associated with an increase in slowly digestible and resistant starch thereby leading to low digestibility (Cai *et al*., 2015; Chung *et al.,* 2011). However, it is not clear whether the desirable attributes of African rice as highlighted above are due to the high AC or some yet‐to‐be‐reported eating and cooking property. It is likely that the AC may not be high enough to increase the resistant starch significantly and therefore influence digestibility. More research is therefore required on the digestibility and other related attributes of African rice compared to Asian rice.

### Analysis of natural variation identifies novel SNPs in African rice *GBSS1*

Though the biparental mapping resulted in the identification of only two nonsynonymous SNPs in *GBSS1* (Table 2), analysis of *O. glaberrima* natural germplasm identified seemingly rare allelic variation in this gene which was not segregating in the RIL population. To our knowledge, this is the first study that has identified such a large number of putative markers in *GBSS1* in African rice. The identification of such high variation in terms of nonsynonymous variants in the *GBSS1* loci is consistent with a recent study in African rice which found a wider diversity of AC than earlier reported (Gayin *et al*., 2015). Previously, African rice has been reported to have a narrow range of AC of about 25% and above (Wang *et al*., 2015; Watanabe *et al*., 2002) but Gayin *et al*. (2015) reported AC ranging from 15.1% to 29.6%. There seems to be no information on the genetic mechanisms underlying this recently discovered variability in AC.

The novel SNP markers identified in this study seem to be of low frequency as majority of them were found in only one of the ten accessions analysed. This low frequency of variants in African rice is consistent with extremely low nucleotide diversity that has been reported in this species (Li *et al*., 2011; Nabholz *et al*., 2014; Orjuela *et al*., 2014; Wang *et al*., 2014) which is suggestive of a severe domestication bottleneck. It is possible that the reason why it has so far not been possible to explain the heritable genetic variation for AC in African rice is because the alleles contributing to this variation are rare as a result of purifying selection (Morrell *et al*., 2012; Teri *et al*., 2009). These alleles may be associated with undesirable starch physicochemical properties and may therefore have been selected against during domestication. These alleles may for example have been associated with high amylose while farmers may have selected for low amylose genotypes. This study brings to the fore the weaknesses of biparental mapping which is the genetic mapping approach that has mostly been used in the analysis of molecular control of AC in African rice. Only a limited amount of diversity is found segregating in biparental populations as compared to use of more diverse germplasm which is likely to have a higher repertoire of alleles. Due to the low frequency of variants in *GBSS* as already highlighted, efforts to identify markers controlling AC in this cultivated species may face challenges as such pedigree‐based populations are likely to miss important alleles. To date only a limited number of African rice accessions have been used in biparental mapping limiting the variation that may have been deployed in genetic mapping. To increase the range of variation that can be surveyed from *GBSS*, the use of association studies is recommend as these provide more power and resolution than structured segregating mapping population. It would be important to include as wide a spectrum of diversity of *O. glaberrima* as possible including the recently reported low AC accessions (Gayin *et al*., 2015).

### Conclusion and implications of this study in breeding for amylose

This study has clearly demonstrated the potential of whole genome‐based BSA in dissecting the genetic basis of complex traits. By conducting genomewide analysis, this study presents the most comprehensive analysis of the genetic architecture of AC in African rice to date. With a global view of the molecular architecture of amylose synthesis in African rice, this integrative genetic analysis has advanced our knowledge of this pathway by providing information on potential transcriptional regulatory pathways. An integrated approach involving analysis of regulatory motifs in gene co‐expression networks is a powerful approach to conduct functional annotation of genes and gain insights on transcriptional regulatory relationships (Sarkar and Maitra 2008; Vandepoele *et al.,* 2009; Yang *et al*., 2015). The genes encoding *NAC* and *CCAAT‐HAP5* TFs may have roles in transcriptionally regulating genes associated with amylose biosynthesis. It is possible that they are involved in the transcriptional regulation of *GBSS1* structural gene.

The candidate genes and markers identified in this study present novel targets for manipulating AC. They can be used to incorporate the potential starch‐related health benefits of African rice into high yielding rice varieties which is likely to result in healthier rice. With *GBSS1* having been identified as the major loci affecting AC, breeding for high amylose rice would entail selecting for the *GBSS1* gene from *O. glaberrima*.

## Experimental procedures

### Mapping population

The analysis presented in this study was conducted on BC_2_F_8_ RIL population developed at Africa Rice Center from an Asian and African rice interspecific cross. CG14, the *O. glaberrima* parent acted as the donor parent while WAB 56–104, the *O. sativa* parent as the recurrent parent (Jones *et al*., 1997). CG14 is an *O. glaberrima* accession from Senegal that has several desirable attributes among them drought resistance and weed competitiveness. It however has low yield potential mainly due to lodging and shattering (Dingkuhn *et al*., 1998; Jones *et al*., 1997). WAB 56–104 is an improved *Japonica* variety that was developed in Africa Rice Center and is characterized by high yields and early maturity (Semagn *et al*., 2007).

### Amylose content determination

Phenotyping for AC was carried out on a total of 100 interspecific progenies together with their parental lines. About 60 seeds were first dehusked and then milled to fine flour using the (QIAGEN tissuelyser, Hilden, Germany). AC was measured using the Megazyme amylose/amylopectin assay kit (Megazyme International Ltd., Bray, Ireland) according to manufacturer's procedure.

### Bulk segregant analysis

Bulk segregant analysis (Michelmore *et al*., 1991) is a rapid and cost‐effective genetic mapping approach that helps identify QTLs, genes and molecular markers controlling target traits. With the advent of cheap next generation sequencing technologies, BSA coupled with whole genome sequencing has increasingly become a popular strategy as it is easy and cheap to develop dense SNP markers across the genome. These dense SNP markers would enable mapping QTLs at finer resolution (Magwene *et al*., 2011). Under this approach, it would be expected that phenotype‐neutral regions would have equal contribution from the two parental genomes while phenotype‐linked regions would show significantly diverged allele frequencies between the two DNA pools.

The two bulks used in this study were constituted by selecting ten individuals each from both extreme ends of the amylose normal frequency distribution curve (Figure 1). The bulk with low AC hereby referred to as ‘LAB’ had individuals with AC of 21.6% and below while the one associated with high AC hereby referred to as ‘HAB’ comprised of individuals with AC of 24.1% and above.

### DNA extraction and quantification

Genomic DNA extraction was conducted from about fifteen 1‐month‐old seedlings using a modified cetyltrimethylammonium bromide (CTAB) protocol that was originally published by Carroll *et al*. (1995). DNA quality was assessed using 0.7% (w/v) gel electrophoresis and NanoDrop 8000 UV‐Vis Spectrophotometers (Thermo Fisher Scientific, Wilmington, DE). DNA quantification was carried out using the Qubit® 3.0 Fluorometer (Life Technologies, Carlsbad, CA) with the dsDNA HS (High Sensitivity) Assay Kit. In order to constitute the two DNA bulks, equal amounts of DNA were bulked and the final concentration adjusted to about 70 ng/μL for both bulks.

### Sequencing and data processing

Genomic DNA libraries were prepared for the pair of DNA pools together with the parental DNA samples. They were then pooled and sequenced in one lane using an Illumina HiSeq sequencer. The sequencing reads were trimmed using CLC Genomic Workbench 9.0 (CLC Bio, a QIAGEN Company, Aarhus, Denmark) using a quality limit of 0.01 (Only 1% of low‐quality bases are allowed). The trimmed reads were then separately mapped against the *O. sativa* Nipponbare reference (IRGSP, 2005) and the *O. glaberrima* CG14 reference (Wang *et al*., 2014) under the following parameters: length fraction 0.5, similarity fraction 0.8, mismatch cost 2, deletion and insertion cost 3. Most read mappers have weaknesses when mapping reads in genomic regions containing indels (Ries *et al*., 2016) and so the read mappings were subjected to two‐pass local realignment using the CLC local realignment tool. This helped remove realignment artefacts and thereby avoid false SNP calls especially near indel positions. Reads exhibiting nonspecific matches were mapped randomly thereby ensuring multiple read alignments were avoided.

**Figure 3:**
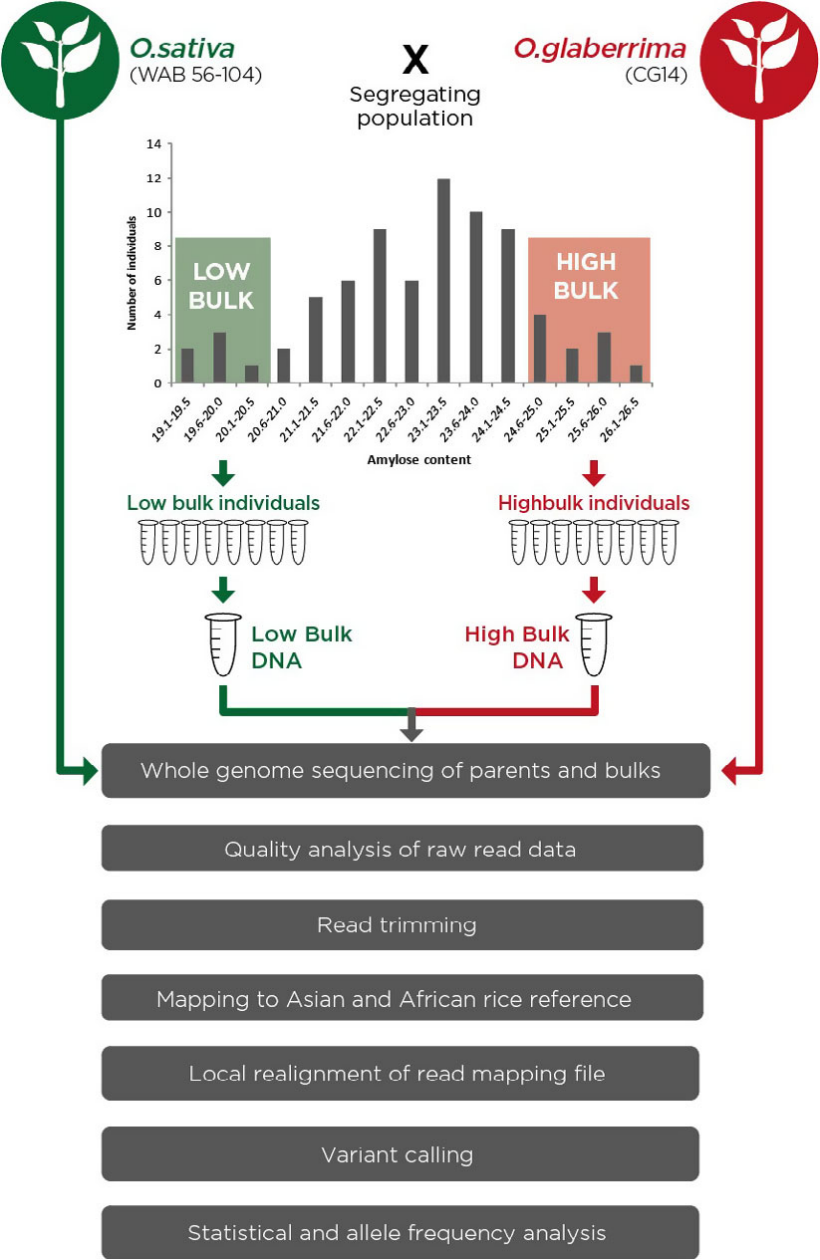
Schematic diagram of bulk segregant analysis experimental set‐up and data analysis pipeline.

SNPs were called using the basic variant detection tool with a minimum coverage of 20, read count of 20 and allele frequency of 90% for the two bulks and a minimum coverage of 5, read count of 5 and allele frequency of 100% for the parents. After SNP calling, a two‐way comparison of SNPs identified in the two bulks was conducted, with SNPs present in both bulks being filtered out. SNPs with a phred score of <30 were filtered out. Additionally, positions with multi‐allelic nucleotide variations as well as those showing nonparental alleles were excluded as these were likely to be false positives which could have been caused by sequencing errors or errors in variant detection. The analysis pipeline that we used is shown in Figure 3.

Analysis for marker‐trait association was then conducted by quantitative evaluation of allele frequencies of the identified nonsynonymous SNPs. The contribution of the parental alleles in the bulked DNA was assessed with the focus being genetic marker sites where the allele from the high amylose parent (African rice) showed over‐representation in the HAB. A cut‐off of 90% allele frequency in the high bulk was used while over‐representation of the alternate allele in the LAB was assessed by a statistical test. The analysis for candidate markers was conducted in two steps. In the first step, the analysis focused on candidate marker positions where the parental genomes were fully homozygous. In the second step, the focus was on genetic marker sites where the parents were heterozygous but the two bulks were fully homozygous for different SNP alleles. Candidate marker positions obtained from this analysis were subject to further statistical analysis.

### Statistical analysis

A chi‐square test was carried out on the allele frequency of the identified SNPs in both bulks separately to assess the cosegregation pattern of the parental alleles in the two bulks. Specifically, the chi‐square test was used to determine the causativeness of the identified SNPs by assessing whether they follow a binomial distribution of 1 : 1 as would be expected of noncausative mutations. Only SNPs that did not follow a binomial distribution of 1 : 1 in both bulks at *P* < 0.05 were retained for further analysis.

### Gene expression and co‐expression analysis

Using the principle of gene co‐expression, the current study sought to identify candidate genes with similar expression patterns with known starch biosynthesis genes. The principle of gene co‐expression is based on the assumption that genes with similar expression patterns are likely to be functionally related. A total of 23 starch synthesis‐related genes (Ohdan *et al.,* 2005) were used as guide genes to conduct gene co‐expression analysis using the Rice Functionally Related gene Expression Network Database (RiceFREND) platform (Sato *et al.,* 2013) and PlantPAN (Chow *et al.,* 2016). RiceFREND is a platform for retrieving co‐expressed gene networks. A Pearson correlation coefficient (PCC) cut‐off of 0.6 or greater was used to identify co‐expressed genes (Aoki *et al.,* 2007; Fu and Xue, 2010). Gene expression profiles for the various co‐expressed genes were obtained from the Rice Expression Profile database (RiceXPro version 3.0). The gene expression profile found in RiceXPro is obtained from microarray data of various tissues at different growth stages (Sato *et al.,* 2011).

### Conserved regulatory motif analysis

The promoter regions of the candidate genes were analysed for conserved regulatory motifs using PlantPAN (The Plant Promoter Analysis Navigator, http://PlantPAN2.itps.ncku.edu.tw) (Chow *et al.,* 2016).

### Analysis of *GBSS1* from unrelated accessions of African rice and *Oryza barthii*

A total of 64 accessions comprising ten of African rice and 54 of *O. barthii* were analysed in this study (Table S4). Whole genome short read data for these samples was downloaded from the short reads archive (SRA) database. The reads were trimmed using CLC Genomic Workbench 9.0 with a quality score limit of 0.01. Read mapping was carried out to the *O. sativa GBSS1* gene sequences using CLC Genomic Workbench 9.0 under the following parameters: length fraction 0.5, similarity fraction 0.8, mismatch cost 2, deletion and insertion cost 3. Variant detection was conducted using the basic variant detection tool of CLC genomics Workbench under the following parameters: minimum coverage 20, read count 8 and allele frequency 30%.

## Author contributions

RH conceived the research idea. RH, PW and AF designed the experiments, PW conducted the experiments, RH supervised the research, MN provided the mapping population, PW wrote the paper with contributions from RH, all authors revised and approved the manuscript.

## Conflict of interest

The authors declare no conflict of interest.

## Supporting information


**Figure S1** Spatial‐temporal expression of granule bound starch synthase 1 (*GBSS1*) (LOC_Os06g04200).
**Figure S2** Spatial‐temporal expression of histone‐fold domain containing protein (LOC_Os01g01290).
**Figure S3** Spatial‐temporal expression of *NAC* Transcription factor (LOC_Os11g31330).
**Table S1** Sequencing statistics.
**Table S2** Statistics of reads uniquely mapped against *O. sativa* and *O. glaberrima* references.
**Table S3** Nonsynonymous candidate SNPs putatively associated with amylose content in African rice.
**Table S4** *O. glaberrima* and *O. barthii* accessions used in the analysis of *GBSS1* gene.
**Table S5** *Cis* regulatory motifs present in the candidate genes.
