# Supplementary material for: Sequencing of bulks of segregants allows dissection of genetic control of amylose content in rice
Source: Plant Biotechnol J. 2017 Jul 11;16(1):100–10. doi: 10.1111/pbi.12752 (PMC5785344; doi:10.1111/pbi.12752)
Supplement: Supplementary file 1 — Figure S1 Spatial‐temporal expression of granule bound starch synthase 1 (GBSS1) (LOC_Os06g04200). Figure S2 Spatial‐temporal expression of histone‐fold domain containing protein (LOC_Os01g01290). Figure S3 Spatial‐temporal expression of NAC Transcription factor (LOC_Os11g31330). Table S1 Sequencing statistics. Table S2 Statistics of reads uniquely mapped against O. sativa and O. glaberrima references. Table S3 Nonsynonymous candidate SNPs putatively associated with amylose content in African rice. Table S4 O. glaberrima and O. barthii accessions used in the analysis of GBSS1 gene. Table S5 Cis regulatory motifs present in the candidate genes. [file PBI-16-100-s001.docx]

**Supplementary information**

**Table S1: Sequencing Statistics**

| **Sample** | **Number of raw reads** | **Sequencing depth^a^** | **Trimmed reads** | |
| --- | --- | --- | --- | --- |
|  |  |  | **Number** | **Average size** |
| HAB | 131,008,138 | 53X | 130,975,209 | 114.9 |
| LAB | 145,429,179 | 59X | 145,393,305 | 115.6 |
| WAB 56-104 | 35,290,472 | 14X | 35,281,191 | 114.3 |
| CG14 | 31,816,416 | 13X | 31,808,913 | 115.8 |

^a^ Average read depth across the genome

**Table S2: Statistics of reads uniquely mapped against *O. sativa* and *O. glaberrima* references**

| **Sample** | ***O. sativa* reference** | | | ***O. glaberrima* reference** | | |
| --- | --- | --- | --- | --- | --- | --- |
|  | **Number** | **%** | **Average coverage** | **Number** | **%** | **Average coverage** |
| HAB | 112021886 | 85.53 | 35.77X | 104532384 | 79.81 | 38.03X |
| LAB | 119224875 | 81.98 | 38.39X | 110919141 | 71.88 | 41.01X |
| WAB 56-104 | 30624443 | 86.78 | 9.53X | 28252141 | 80.08 | 10.30X |
| CG14 | 29729099 | 93.44 | 9.82X | 28756017 | 90.4 | 10.85X |

*Percentage of the genome covered by the short reads. The genome size of the two bulks was estimated to be equivalent to that of *O. sativa* (370Mb) since it is assumed that the successive backcrosses during mapping population development had recovered a substantial portion of the *O. sativa* genome

**Table S3: Non-synonymous candidate SNPs putatively associated with amylose content in African rice**

| **Locus ID** | **Gene name** | **SNP** | **Position** | **Amino acid change** |
| --- | --- | --- | --- | --- |
| LOC_Os06g04040 | WD domain, G-beta repeat domain containing protein | T/C | 1653659 | Ser to Pro |
| LOC_Os06g04060 | expressed protein | T/G | 1693774 | Ser to Ala |
| LOC_Os06g04169 | hydrolase, alpha/beta fold family domain containing protein | G/C | 1736462 | Thr to Ser |
| LOC_Os06g04200 | Granule-bound starch synthase 1 | G/A | 1769686 | Asp to Asn |
| LOC_Os06g04280 | 3-phosphoshikimate 1-carboxyvinyltransferase, chloroplast precursor | C/T | 1816306 | Ala to Val |
| LOC_Os06g04440 | expressed protein | G/A | 1900141 | Cys to Tyr |
| LOC_Os06g04520 | WASH complex subunit 7-like isoform X1 | T/C | 1945370 | Phe to Ser |
| Loc_Os06g04550 | expressed protein | T/C | 1971594 | Ser to Pro |
| Loc_Os06g04550 | expressed protein | T/C | 1971601 | Leu to Pro |
| LOC_Os06g04660 | oxidoreductase, 2OG-Fe oxygenase family protein | A/T | 2030977 | Lys to Met |
|  |  | T/C | 2030920 | Leu to Pro |
|  |  | A/T | 2030977 | Lys to Met |
|  |  | C/G | 2031158 | Ile to Met |
|  |  | A/C | 2032835 | Lys to Thr |
| LOC_Os06g04790 | HAD superfamily phosphatase | G/T | 2088936 | Glu to Asp |
| LOC_Os06g04850 | homeobox associated leucine zipper | G/A | 2123781 | Val to Met |
|  |  | A/G | 2123811 | Thr to Ala |
| LOC_Os06g04970 | serine protease-like | C/T | 2193215 | Thr to Ile |
|  |  | T/C | 2189916 | Trp to Arg |
|  |  | A/T | 2191680 | Lys to Met |
|  |  | T/C | 2193139 | Ser to Pro |
|  |  | C/T | 2193215 | Thr to Ile |
| LOC_Os06g05050 | OsWAK61 - OsWAK receptor-like protein kinase | A/G | 2231063 | Lys to Glu |
|  |  | A/C | 22311069 | Ile to Leu |
|  |  | G/A | 2232026 | Asp to Asn |
|  |  | G/C | 2232134 | Asp to His |
|  |  | G/A | 2232429 | Ser to Asn |
|  |  | A/G | 2232713 | Thr to Ala |
|  |  | A/G | 2232827 | Thr to Ala |
|  |  | T/G | 2232834 | Met to Arg |
|  |  | C/T | 2232866 | Leu to Phe |
|  |  | G/A | 2232890 | Val to Met |
|  |  | G/C | 2232980 | Glu to Gln |
| LOC_Os06g05130 | myristoyl-acyl carrier protein thioesterase, chloroplast precursor | G/A | 2278677 | Ala to Thr |
| LOC_Os06g05220 | expressed protein | T/G | 2342262 | Ser to Ala |
| Loc_Os06g05230 | retrotransposon protein | T/G | 2345081 | Asp to Glu |
|  |  | A/G | 2345508 | Ile to Val |
|  |  | G/A | 2345643 | Gly to Arg |
|  |  | G/A | 2346661 | Gly to Glu |
|  |  | A/G | 2347278 | Ser to Gly |
|  |  | A/G | 2347621 | His to Arg |
|  |  | C/G | 2347638 | Gln to 2Glu |
|  |  | G/A | 2347773 | Glu to Lys |
|  |  | T/C | 2348010 | Cys to Arg |
|  |  | A/G | 2348301 | Ile to Val |
|  |  | T/C | 2348304 | Cys to Arg |
| LOC_Os06g05260 | pectate lyase precursor, putative | T/G | 2373497 | Leu to Arg |
|  |  | T/A | 2373890 | Met to Lys |
|  |  | G/A | 2374173 | Arg to His |
| Loc_Os06g05284 | transferase family protein | A/G | 2381841 | Asn to Asp |
| Loc_Os06g05310 | transferase family protein | A/G | 2389627 | Lys to Glu |
| LOC_Os06g05350 | whirly transcription factor domain containing protein | G/T | 2405572 | Ala to Ser |
| LOC_Os06g05359 | NBS-LRR disease resistance protein | C/A | 2414037 | Ser to Arg |
|  |  | A/G | 2415254 | His to Arg |
|  |  | A/C | 2415341 | His to Pro |
|  |  | T/G | 2415448 | Trp to Gly |
|  |  | A/G | 2416555 | Asn to Asp |
| LOC_Os06g05368 | expressed protein | A/G | 2419603 | Lys to Arg |
| LOC_Os06g05380 | WRKY73 | C/T | 2429308 | Leu to Phe |
|  |  | T/C | 2433127 | Leu to Pro |
| LOC_Os06g05450 | expressed protein | T/C | 2461309 | Leu to Pro |
| LOC_Os06g05540 | expressed protein | A/G | 2510507 | His to Arg |
|  |  | C/T | 2510549 | Ala to Val |
| Loc_Os06g05580 | OsFBDUF30 - F-box and DUF domain containing protein | G/C | 2530751 | Met to Ile |
| LOC_Os06g05590 | OsFBX186 - F-box domain containing protein | T/C | 2533567 | Leu to Pro |
| LOC_Os06g05600 | OsFBDUF31 - F-box and DUF domain containing protein | C/G | 2536081 | Arg to Gly |
|  |  | T/C | 2564466 | Met to Thr |
| Loc_Os06g05610 | OsFBDUF32 - F-box and DUF domain containing protein | G/A | 2538835 | Gly to Asp |
|  |  | C/T | 2538859 | Ala to Val |
|  |  | C/G | 2539218 | Leu to Val |
| LOC_Os06g05620 | OsFBDUF33 - F-box and DUF domain containing protein | C/T | 2543338 | Arg to Cys |
| LOC_Os06g05630 | GDSL-like lipase/acylhydrolase | G/A | 2548150 | Arg to Gln |
|  |  | T/C | 2548180 | Ile to Thr |
|  |  | T/C | 2548862 | Leu to Ser |
| LOC_Os06g05690 | cysteine synthase, chloroplast/chromoplast precursor | G/A | 2574901 | Val to Ile |
| LOC_Os06g05710 | pollen-specific protein | C/A | 2582951 | Ala to Glu |
| LOC_Os06g05760 | ubiquitin family protein | A/G | 2608688 | Arg to Gly |
| LOC_Os06g05820 | OsLonP2 - Putative Lon protease homologue | A/G | 2654150 | Ile to Val |
| LOC_Os06g05870 | dual specificity protein phosphatase | C/G | 2688014 | Pro to Ala |
| LOC_Os06g06080 | serine esterase family protein | G/A | 2804399 | Gly to Glu |
| Loc_Os06g06080 | serine esterase family protein | G/C | 2803491 | Ser to Thr |
| LOC_Os06g06250 | GDSL-like lipase/acylhydrolase | C/T | 2895517 | Pro to Ser |
| LOC_Os06g06320 | osFTL2 FT-Like2 homologous to Flowering Locus T gene; contains Pfam profile PF01161: Phosphatidylethanolamine-binding protein | G/C | 2940248 | Lys to Asn |
| LOC_Os06g06330 | expressed protein | A/T | 2945721 | His to Leu |
| LOC_Os06g06350 | AMP-binding enzyme | A/G | 2954475 | Ile to Met |
| LOC_Os06g06430 | expressed protein | T/A | 3000187 | Val to Glu |
| LOC_Os06g06450 | heat shock protein STI | A/C | 3036142 | Glu to Asp |
|  |  | T/A | 3038682 | Val to Asp |
|  |  | C/G | 3039165 | His to Gln |
|  |  | T/C | 3039205 | Phe to Leu |
| LOC_Os06g06470 | U-box domain containing heat shock protein | C/T | 3042434 | Thr to Ile |
|  |  | T/A | 3043759 | Ser to Thr |
|  |  | G/A | 3045525 | Glu to Lys |
| LOC_Os06g06540 | AP2 domain containing protein | T/G | 3068018 | Ser to Ala |
|  |  | C/A | 3068049 | Ala to Glu |
|  |  | C/T | 3068052 | Ala to Val |
| LOC_Os06g06740 | MYB family transcription factor | T/A | 3162015 | Val to Asp |
| LOC_Os06g06770 | OsPOP11 - Putative Prolyl Oligopeptidase homologue | G/A | 3186805 | Arg to His |
| LOC_Os06g06790 | OsPDIL1-5 protein disulfide isomerase PDIL1-5 | G/T | 3200117 | Ser to Ile |
| LOC_Os06g07690 | expressed protein | G/A | 3721726 | Ser to Asn |
| LOC_Os06g07700 | expressed protein | C/T | 3723446 | Arg to Trp |
| LOC_Os06g07770 | expressed protein | C/T | 3764827 | Ser to Phe |
|  |  | C/T | 3770955 | His to Tyr |
| LOC_Os06g07790 | retrotransposon protein | C/T | 3784973 | Leu to Phe |
| LOC_Os06g07800 | expressed protein | G/T | 3788267 | Cys to Phe |
| LOC_Os06g07810 | transposon protein, putative, unclassified | G/A | 3794599 | Gly to Asp |

**Table S4: *O. glaberrima* and *O. barthii* accessions used in the analysis of *GBSS1* gene**

| **Species** | **Genebank accession number^1^** | **GenBank accession number^2^** | **Origin** |
| --- | --- | --- | --- |
| *O. glaberrima* | IRGC103469 | SRX502298 | Burkina Faso |
| *O. glaberrima* | TOG5923 | SRX502301 | Liberia |
| *O. glaberrima* | TOG5949 | SRX502302 | Liberia |
| *O. glaberrima* | IRGC103472 | SRX502306 | Burkina Faso |
| *O. glaberrima* | IRGC103632 | SRX502308 | Mali |
| *O. glaberrima* | IRGC104574 | SRX502311 | Mali |
| *O. glaberrima* | IRGC104955 | SRX502312 | Sierra Leone |
| *O. glaberrima* | CG14 |  |  |
| *O. glaberrima* | IRGC104040 |  | Chad |
| *O. glaberrima* | IRGC 101328 |  | Sierra Leone |
| *O. barthii* | IRGC 103590 |  | Cameroon |
| *O. barthii* | IRGC 101381 |  | Niger |
| *O. barthii* | IRGC 104124 |  | Chad |
| *O. barthii* | IRGC 86481 |  | Zambia |
| *O. barthii* | IRGC100122 | SRX502162 | Gambia |
| *O. barthii* | IRGC100934 | SRX502164 | Mali |
| *O. barthii* | WAB0009240 | SRX502191 | Cameroon |
| *O. barthii* | WAB0012712 | SRX502192 | Mali |
| *O. barthii* | WAB0024904 | SRX502193 | Nigeria |
| *O. barthii* | WAB0026768 | SRX502194 | Unknown |
| *O. barthii* | WAB0026769 | SRX502195 | Nigeria |
| *O. barthii* | WAB0026770 | SRX502196 | Nigeria |
| *O. barthii* | WAB0028874 | SRX502197 | Gambia |
| *O. barthii* | WAB0028876 | SRX502199 | Guinea |
| *O. barthii -III* | WAB0009239 | SRX502190 | Nigeria |
| *O. barthii* | WAB0028952 | SRX502173 | Zambia |
| *O. barthii* | WAB0028927 | SRX502223 | Chad |
| *O. barthii* | WAB0028992 | SRX502179 | Chad |
| *O. barthii* | WAB0028937 | SRX502228 | Nigeria |
| *O. barthii* | WAB0028938 | [SRX502172](http://www.ncbi.nlm.nih.gov/sra/SRX502172%5baccn%5d) | Nigeria |
| *O. barthii* | WAB0028940 | SRX502229 | Nigeria |
| *O. barthii* | WAB0028875 | [SRX502198](http://www.ncbi.nlm.nih.gov/sra/SRX502198%5baccn%5d) | Mali |
| *O. barthii* | WAB0028980 | SRX502177 | Mali |
| *O. barthii* | WAB0028975 | SRX502241 | Mali |
| *O. barthii* | WAB0028946 | SRX502232 | Cameroon |
| *O. barthii* | WAB0028981 | SRX502243 | Mali |
| *O. barthii* | WAB0028897 | SRX502209 | Mali |
| *O. barthii* | WAB0028916 | SRX502218 | Mali |
| *O. barthii* | WAB0028931 | SRX502226 | Chad |
| *O. barthii* | IRGC103534 | SRX502189 | Mali |
| *O. barthii* | IRGC101240 | SRX502185 | Mali |
| *O. barthii* | IRGC100931 | SRX502163 | Mali |
| *O. barthii* | IRGC104119 | SRX502167 | Chad |
| *O. barthii* | IRGC105608 | SRX502168 | Cameroon |
| *O. barthii* | WAB0028877 | SRX502200 | Niger |
| *O. barthii* | IRGC103912 | SRX502170 | Tanzania |
| *O. barthii* | WAB0028903 | [SRX502171](http://www.ncbi.nlm.nih.gov/sra/SRX502171%5baccn%5d) | Zambia |
| *O. barthii* | WAB0028934 | SRX502227 | Chad |
| *O. barthii* | WAB0028942 | SRX502230 | Cameroon |
| *O. barthii* | WAB0029000 | SRX502253 | Botswana |
| *O. barthii* | WAB0028917 | SRX502219 | Chad |
| *O. barthii* | WAB0028926 | SRX502222 | Chad |
| *O. barthii* | WAB0028929 | SRX502224 | Chad |
| *O. barthii* | IRGC106234 | SRX502169 | Sierra Leone |
| *O. barthii* | WAB0028987 | SRX502178 | Nigeria |
| *O. barthii* | WAB0028910 | SRX502213 | Mali |
| *O. barthii* | WAB0028911 | SRX502214 | Mali |
| *O. barthii* | WAB0028912 | SRX502215 | Mali |
| *O. barthii* | IRGC100922 | SRX502182 | Unknown |
| *O. barthii* | WAB0028893 | SRX502206 | Mali |
| *O. barthii* | WAB0028900 | SRX502210 | Mali |
| *O. barthii* | WAB0028913 | SRX502216 | Mali |
| *O. barthii* | IRGC100927 | SRX502183 | Sierra Leone |
| *O. barthii* | WAB0030186 | SRX502255 | Mali |
| *O. barthii* | WAB0028915 | SRX502217 | Mali |

^1^Refers to the unique identifier used by the genebank where it was sourced from. Samples with Accession numbers starting with TOG were obtained from Africa Rice Center while those starting with IRGC were obtained from IRRI

^2^ Refers to unique sequence record identifier for the GenBank’s SRA database

Modified from ([Wang et al. 2014](#_ENREF_57))

**Table S5: *Cis* regulatory motifs present in the candidate genes**

| **Sl.No** | **Motif name** | ***cis* element** | **Function** | **Reference** |
| --- | --- | --- | --- | --- |
| 1 | AACACOREOSGLUB1 | AACAAAC | Core of AACA motifs found in rice (O.s.) glutelin genes. Required in combination with GCN4 and ACGT to confer endosperm and seed specific expression | (Washida et al. 1999; Wu et al. 2000) |
| 2 | ABREMOTIFIOSRAB16B | AGTACGTGGC | Motif required for ABA responsiveness | (Ono et al. 1996) |
| 3 | ACGTOSGLUB1 | GTACGTG | ACGT motif found in GluB-1 gene in rice. Interacts with GCN4, AACA and ACGT to confer endosperm expression | (Washida et al. 1999; Wu et al. 2000) |
| 4 | GLUTEBP1OS | AAGCAACACACAAC | Binding site in the promoter region of glutelinGt3 gene |  |
| 5 | PROLAMINBOXOSGLUB1 | TGCAAAG | Involved in quantitative regulation of the GluB-1 gene | (Wu et al. 2000) |
| 6 | CCAAT-Box | CCAAT | Involved in multiple pathways including regulation of seed specific expression | (Laloum et al. 2012) |
| 7 | GLUTAACAOS | AACAAACTCTAT | glutelin common motif; "AACA motif | (Takaiwa and Oono 1990) |
| 8 | GT1CONSENSUS | GRWAAW | binding site in many light-regulated genes, |  |


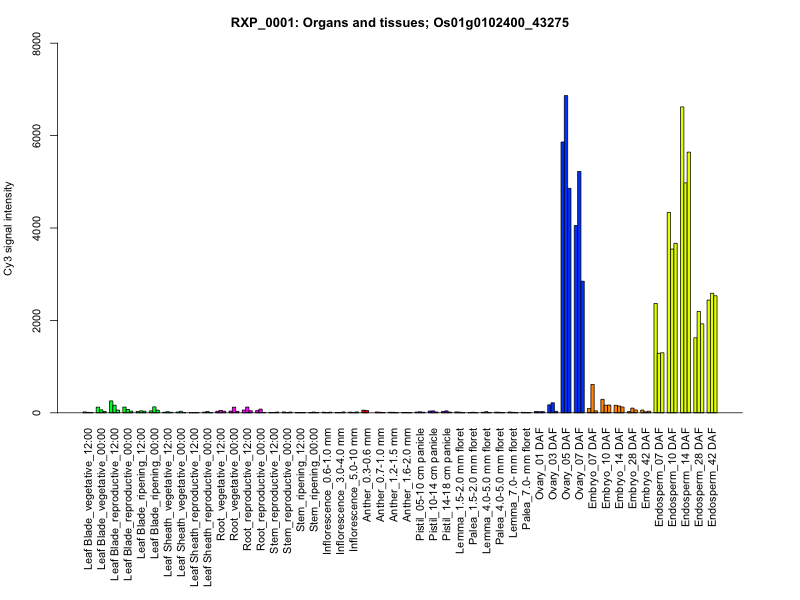


**Supplementary Figure 1: Spatial-temporal expression of granule bound starch synthase 1 (*GBSS1*) (LOC_Os06g04200)**

**
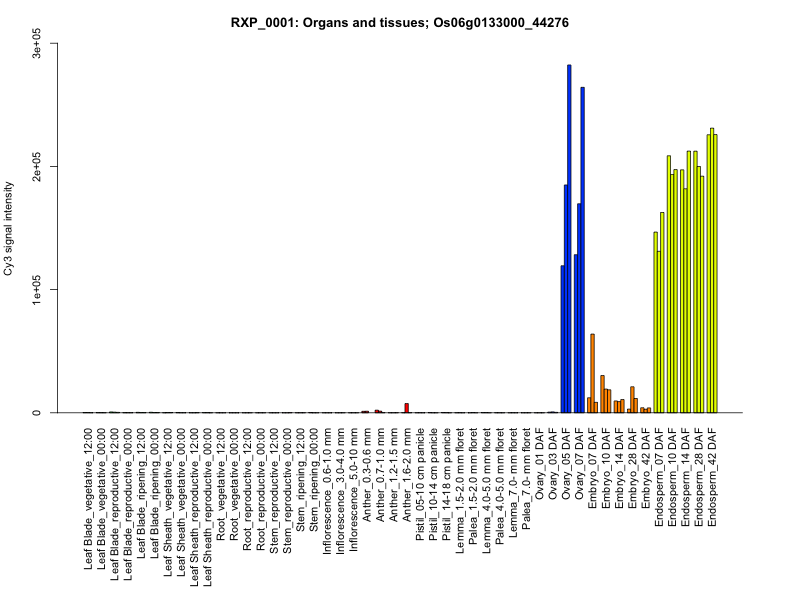
**

**Supplementary Figure 2: Spatial-temporal expression of histone-fold domain containing protein (LOC_Os01g01290)**


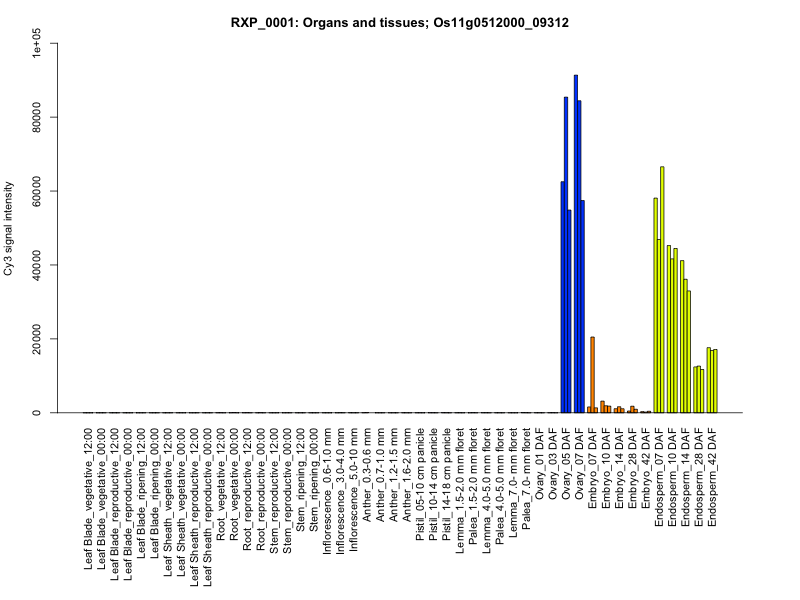


**Supplementary Figure 3: Spatial-temporal expression of *NAC* Transcription factor (LOC_Os11g31330)**
